# Neurocognitive working mechanisms of the prevention of relapse in remitted recurrent depression (NEWPRIDE): protocol of a randomized controlled neuroimaging trial of preventive cognitive therapy

**DOI:** 10.1186/s12888-019-2384-0

**Published:** 2019-12-19

**Authors:** Rozemarijn S. van Kleef, Claudi L. H. Bockting, Evelien van Valen, André Aleman, Jan-Bernard C. Marsman, Marie-José van Tol

**Affiliations:** 1 Cognitive Neuroscience Center, Department of Biomedical Sciences of Cells and Systems, University Medical Center Groningen, University of Groningen, Antonius Deusinglaan 2, 9713 AW Groningen, The Netherlands; 2 Department of Psychiatry and Urban Mental Health Institute, Amsterdam University Medical Center, Location AMC, Meibergdreef 9, 1105 AZ Amsterdam, The Netherlands; 3 Department of Geriatrics, Heidelberglaan 100, University Medical Center Utrecht, 3584 CX Utrecht, The Netherlands

**Keywords:** Major depressive disorder, Recurrence, Remission, Prevention, Randomized controlled trial, Functional neuroimaging, Neurocognitive mechanisms, Therapy prediction

## Abstract

**Background:**

Major Depressive Disorder (MDD) is a psychiatric disorder with a highly recurrent character, making prevention of relapse an important clinical goal. Preventive Cognitive Therapy (PCT) has been proven effective in preventing relapse, though not for every patient. A better understanding of relapse vulnerability and working mechanisms of preventive treatment may inform effective personalized intervention strategies. Neurocognitive models of MDD suggest that abnormalities in prefrontal control over limbic emotion-processing areas during emotional processing and regulation are important in understanding relapse vulnerability. Whether changes in these neurocognitive abnormalities are induced by PCT and thus play an important role in mediating the risk for recurrent depression, is currently unclear.

In the Neurocognitive Working Mechanisms of the Prevention of Relapse In Depression (NEWPRIDE) study, we aim to 1) study neurocognitive factors underpinning the vulnerability for relapse, 2) understand the neurocognitive working mechanisms of PCT, 3) predict longitudinal treatment effects based on pre-treatment neurocognitive characteristics, and 4) validate the pupil dilation response as a marker for prefrontal activity, reflecting emotion regulation capacity and therapy success.

**Methods:**

In this randomized controlled trial, 75 remitted recurrent MDD (rrMDD) patients will be included. Detailed clinical and cognitive measurements, fMRI scanning and pupillometry will be performed at baseline and three-month follow-up. In the interval, 50 rrMDD patients will be randomized to eight sessions of PCT and 25 rrMDD patients to a waiting list. At baseline, 25 healthy control participants will be additionally included to objectify cross-sectional residual neurocognitive abnormalities in rrMDD. After 18 months, clinical assessments of relapse status are performed to investigate which therapy induced changes predict relapse in the 50 patients allocated to PCT.

**Discussion:**

The present trial is the first to study the neurocognitive vulnerability factors underlying relapse and mediating relapse prevention, their value for predicting PCT success and whether pupil dilation acts as a valuable marker in this regard. Ultimately, a deeper understanding of relapse prevention could contribute to the development of better targeted preventive interventions.

**Trial registration:**

Trial registration: Netherlands Trial Register, August 18, 2015, trial number NL5219.

## Background

### Rationale

Major Depressive Disorder (MDD) is the most prevalent psychiatric disorder, with a lifetime prevalence of 19% [1] and a highly recurrent nature [2, 3]. History of recurrence is an important predictor of relapse [4, 5], making prevention of relapse early in the course of the disease an important clinical goal. Understanding the mechanisms facilitating relapse can give insight into the core processes essential for relapse prevention, and may provide markers to guide clinicians in selecting preventive strategies [6].

One way of gaining a better understanding of relapse vulnerability is investigating the neurocognitive mechanisms of existing therapeutic interventions that proved effective in preventing relapse [7, 8]. Clinically, cognitive therapy during the depressive episode has been shown to have an enduring preventive effect [9–11]. Applying preventive cognitive therapy (PCT; a cognitive-therapy based psychological intervention) in the remitted state has shown effectivity in lowering relapse-risk up to 10 years, compared to both no therapeutic interventions and to (tapering) maintenance antidepressant use [12–17]. Studying the working mechanisms of PCT can provide insight into which cognitive and affective processes put an individual at risk for relapse, and which changes therein mediate a lowered vulnerability risk following treatment.

Studies in the acute phase of MDD have shown that cognitive therapy affects neurocognitive functioning, including lowering cognitive biases [18, 19] and increasing prefrontal cortical control over limbic structures during emotional processing [20–22]. These processes are thought to lay at the core of the pathophysiology of MDD [23–29], and might add to the development and perpetuation of depression through overrepresentation and overinterpretation of negative information and negative affect [23, 30–33]. Several studies have shown that abnormalities in the prefrontal cortex persist in the remitted phase of MDD [34–37] and may predict disease course [38–43]. Furthermore, abnormal prefrontal regulation has been related to specific MDD typical cognitive processes [23, 44–49] that may persist after remission and have been linked to recurrence, such as cognitive biases towards negative information [49–51], heightened cognitive reactivity to stressful situations [52–54], negative rumination [55–58], affective reactivity [59], and inadequate emotion regulation (reflected in an increased tendency to engage in, and difficulty to disengage from, negative mood states) [32, 60, 61]. Whether the protective effect of PCT is obtained via alternations in these neurocognitive processes and how individual differences therein hamper such effect is yet unknown.

Though often neglected, difficulties in processing reward and maintaining positive emotions may similarly contribute to relapse vulnerability in MDD. Abnormalities in processing positive emotions have been consistently associated with MDD, also in the remitted phase [37, 62–68]. Moreover, neural responsivity in regions important for reward processing has been related to a history of depressive episodes [69] and both psychological and pharmacological treatment response [70]. In acute MDD, difficulties sustaining positive emotions have been suggested to reflect reduced fronto-striatal capacity [71, 72]. In remitted MDD, Matsubara and colleagues [73] found abnormal fronto-limbic activity during effortful regulation of positive emotions, while others did not [61, 74]. Whether PCT obtains part of its preventive effects by impacting neurocognitive processing of positive emotional material, is not yet known.

### Aims

In the Neurocognitive Working Mechanisms of the Prevention of Relapse In Depression (NEWPRIDE) study, the neurocognitive mechanisms of preventive therapy will be investigated using a within-subject longitudinal comparison of cognitive biases and fMRI characteristics related to positive and negative emotion processing before and after PCT, as compared to a waiting list control group. At baseline, a healthy control (HC) group will be included for cross-sectional comparison of residual abnormalities.

The present study has four main aims. Firstly, we aim to cross-sectionally examine whether cognitive biases and functional magnetic resonance imaging (fMRI) responses during the regulation of positive and negative emotions in medication-free, highly recurrent, remitted MDD (rrMDD) patients differ from controls. We hypothesize residual abnormalities in rrMDD patients compared to HC in (i) an amygdala-insular-subgenual anterior cingulate cortex (ACC)-ventrolateral prefrontal cortex (PFC) circuitry associated with biased processing of negative emotional information, (ii) a striatal-medial PFC circuitry associated with biased processing of positive emotional information, and (iii) the lateral-and medial PFC circuitry associated with cognitive control [75].

Secondly, this randomized controlled fMRI-study is the first to investigate the neurocognitive working mechanisms of PCT (compared to a waiting list condition) in rrMDD patients. We hypothesize that PCT will result in increased lateral and medial prefrontal activation, dampened activation of limbic regions, and improved connectivity between these regions during emotion regulation, which will coincide with normalised processing and regulation of negative information and a lowered likelihood of a prevailing negative mood. Furthermore, we hypothesize that increased PFC activation following PCT relates to increased preferential processing of positive information [70, 76].

Thirdly, we aim to identify pre-treatment neurocognitive markers predictive of long-term PCT success measured at 18-month follow-up. It is expected that low pre-treatment insular and PFC activation [39, 77] and low PFC connectivity with emotion processing areas during emotion processing predicts favourable treatment response [78]. Also, we hypothesize that participants with larger pre-post differences on neurocognitive measures, will show lowest relapse up until18-month follow-up.

Finally, we aim to investigate the value of the pupil dilation response (PDR) as a new predictor of frontal regulatory efforts during emotion regulation and PCT effects as a means of providing cheaper, non-imaging, yet imaging-informed, neurocognitive markers of treatment-success in rrMDD [79–81]. We hypothesize that increased PFC activation following PCT will be reflected in an increased PDR during emotion regulation, and that low pre-treatment PDR-response during emotional regulation will be predictive of PCT effects.

## Methods/design

The NEWPRIDE study is funded by the Dutch Research Council (NWO/ZonMW grant 016.156.077) and the Dutch Brain Foundation (Hersenstichting, Fellowship number F2014(1)-21). The study has been approved by the medical ethical board of the University Medical Center Groningen (2015.284) and is in accordance with the latest version of the Declaration of Helsinki.

### Design

**Fig. 1:**
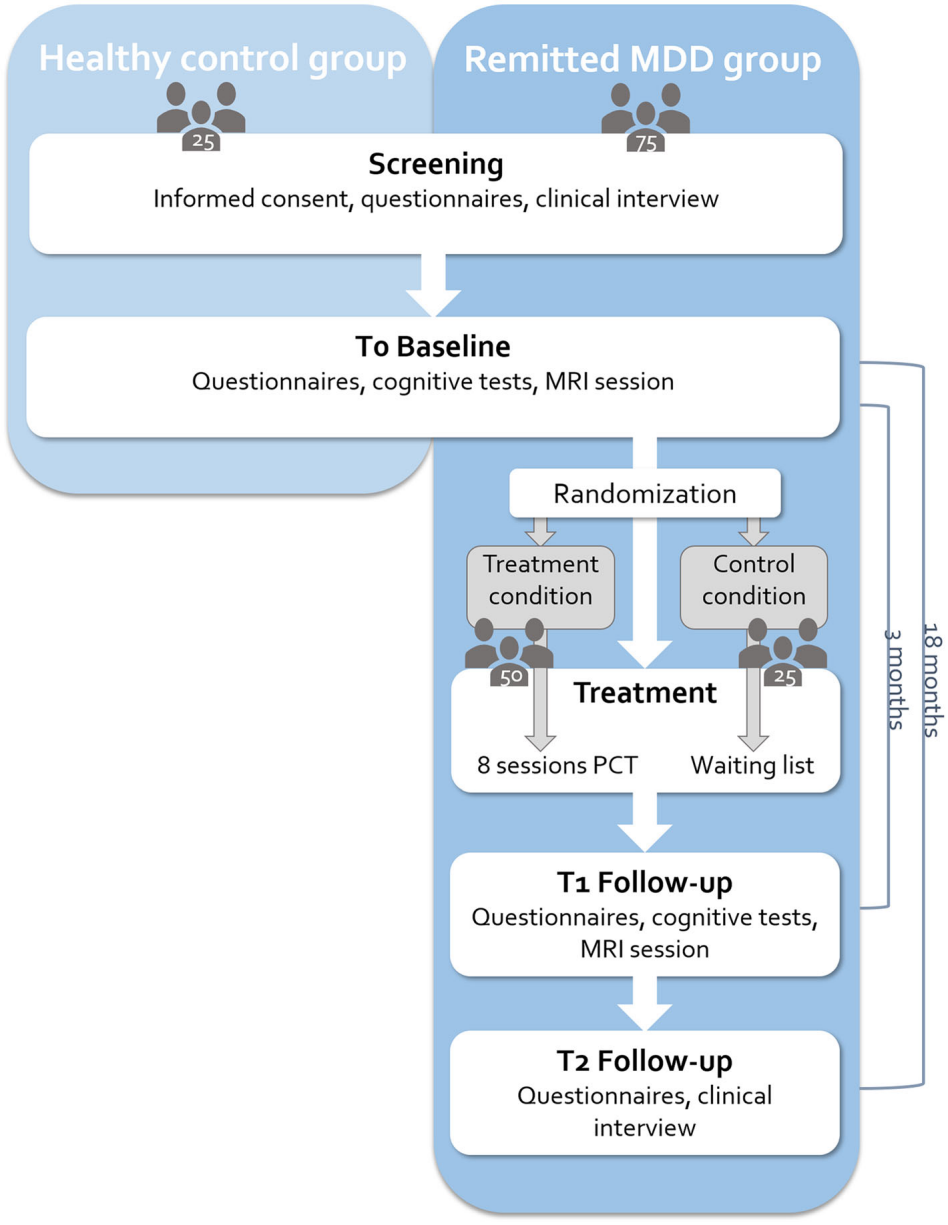
Flowchart providing an overview of the NEWPRIDE study design

NEWPRIDE is an open-label randomized controlled trial (RCT), consisting of four phases following an initial screening: [1] a baseline clinical, neuropsychological, fMRI- and PDR-examination (T0) [2]; a three-month treatment phase, including either eight sessions PCT or a waiting-list control period [3]; a post-treatment clinical, neuropsychological, fMRI- and PDR-examination (T1) 3 months after baseline, and [4] a follow-up clinical examination (T2) 18 months after baseline. A flowchart of the study design is provided in Fig. 1.

### Participants

#### Recruitment

For this study, 75 rrMDD patients will be recruited, plus 25 HC participants, matched for age, sex, and education level. We will recruit rrMDD patients who have been in remission for over 2 months and who are highly recurrent (meaning having experienced two or more depressive episodes in the past 5 years). Given that this population is often no longer in care after remission, we will primarily recruit via advertising and (social) media.

#### Inclusion and exclusion criteria

Criteria for inclusion for all participants are:

- Age 18 to 60 years;
- Normal intelligence (IQ > 85), as assessed with the Dutch Adult Reading Test, or indicated by having finished an education on at least vocational level;
- (Near) native Dutch language proficiency;
- No current DSM-IV diagnosis, according to the Structured Clinical Interview for DSM-IV Axis I disorders (SCID-I);
- No current depressive symptomatology, as indicated by a score of 13 or less on the Inventory of Depressive Symptomatology (IDS-SR) at the time of inclusion;
- No past or present alcohol or drug dependency;
- No general MRI contra-indications.

We apply the following criteria specific for the rrMDD group:

- Meeting the lifetime criteria of a DSM-IV MDD diagnosis, according to the SCID-I;
- Currently in remission from the last Major Depressive Episode (MDE) for more than 2 months, but not longer than 2 years, according to the DSM-IV criteria;
- At least two MDE’s in the past 5 years;
- No regular use of psychotropic medication, including anti-depressant medication, for at least 4 weeks;
- No cognitive (behavioural) therapy for the last MDE;
- No current or past psychotic or manic/hypomanic episode, nor any DSM-IV developmental disorder diagnosis.

Finally, an additional criterion for HC participants is:

- Absence of a lifetime diagnosis of any DSM-IV disorder, as assessed with the SCID-I.

#### Sample size

A total of 100 participants will be included, of which 75 rrMDD patients and 25 HCs. Power analyses for behavioural data, performed with G*Power 3.9.1.4, show that groups of 25 are sufficient to detect moderate effects (with 80% power and α = 0.05) for both cross-sectional group comparisons and longitudinal treatment effect analyses. Exact power analyses for fMRI analyses are difficult, due to the complex mass univariate nature of the data. However, previous comparable imaging studies yielded sufficient power with the inclusion of 20 participants per group [21, 37, 64, 70, 78, 82, 83]. We will include a minimum of 25 (carefully selected) participants per group, to allow for loss of data due to follow-up drop-outs.

The inclusion of 75 rrMDD patients allows for several lines of analysis. Firstly, cross-sectional comparisons will be carried out in 50 rrMDD patients versus 25 HCs in order to establish residual abnormalities in emotion processing. Because of an (unforeseen at the time of planning of the study) replacement of the MRI scanner, the last included 25 rrMDD patients (all allotted to the treatment condition) will be scanned on a different scanner, and will therefore not be included in this analysis. Secondly, longitudinal analyses of immediate treatment effects will be performed in the first 50 rrMDD patients who were randomized to either the therapy group or the waiting list control (25 vs. 25). Finally, since it is expected that 50% of rrMDD patients relapses within 1,5 years [41, 59], an additional group of 25 rrMDD patients will be included for the PCT condition, expanding the group of participants receiving treatment to *n* = 50 to allow analyses of pre-post differences in relation to clinical outcome at 18-month follow-up. Even though these last 25 patients will be scanned on a different MRI scanner, we will ensure that all participants have their pre- and post-treatment scanning session on the same MR machine.

### Intervention

The participants in the treatment condition will receive eight individual face-to-face sessions of PCT, a therapy based on the cognitive model of Beck [84], developed specifically to prevent relapse in remitted MDD patients. The main elements of PCT are (i) identifying and challenging dysfunctional attitudes, (ii) internalising more helpful attitudes, (iii) enhancing the formation of specific memories of positive events, and (iv) formulating future relapse prevention strategies [6, 85].

PCT is provided by experienced and accredited psychologists, fully trained in cognitive behavioural therapy, who have received an additional two-day training in delivering PCT in the context of this study (by EvV and CLHB). To establish an adequate level of treatment integrity, therapists will strictly follow a treatment manual [85] and will be supervised by a cognitive behavioural therapy supervisor (EvV). Finally, the treatment sessions will be audio-recorded to allow reviewing by the supervisor and researchers (only when participants give their permission).

### Measures

#### Primary outcome measures

The primary outcome of the cross-sectional assessment of rrMDD characteristics is threefold: it concerns baseline characteristics at T0 in (i) cognitive biases to negative and positive emotional information (measured with the Attentional Response to Distal vs. Proximal Emotional Information task (ARDPEI) [86], an adapted version of the Emotional Reasoning Task [87] and an Implicit Association Task (IAT) [88]); in (ii) blood oxygenation level dependent (BOLD) response (during an Emotion Regulation Task (ERT) (similar as in [81, 89]), a Verbal Working Memory Task (VWMT) [90], and during resting state; and in (iii) the PDR during these neurocognitive tasks.

Changes in these measures following PCT at T1 are the main study parameters in the assessment of the working mechanisms of PCT. The primary outcome for the assessment of treatment predictors concerns (i) depressive symptomatology, as measured with the Inventory of Depressive Symptomatology self-report version (IDS –SR30) [91] at T0, T1 and T2, and (ii) time to relapse and number of relapse over the course of the study, as measured with the Structured Clinical Interview for DSM-IV disorders (SCID-I) [92] at T1 and T2, in combination with the life chart method at T2.

#### Secondary outcome measures

Secondary parameters concern the following measures at T0 and changes therein following therapy (at T1), as these measurements provide additional information to interpret and understand the changes in neurocognitive functioning: Positive and Negative Affect Scale (PANAS) [93], Domains and Dimensions of Pleasure Scale (DDOPS) [94], Leuven Adaptation of the Rumination on Sadness Scale (LARSS) [95], Emotion Regulation Questionnaire (ERQ) [96], Responses on Positive Affect questionnaire (RPA) [97], Dysfunctional Attitude Scale form A (DAS-A) [98], NEO Five Factor Inventory (NEO-FFI) [99], Leiden Index of Depression Sensitivity–2nd revision (LEIDS-RR) [100], Bermond-Vorst Alexithymia Questionnaire (BVAQ) [101], and Wechsler Adult Intelligence Scale-IV (WAIS-IV) subtests (digit-span, letter-number sequencing, and digit-symbol substitution) [102].

Other study parameters that will be measured during MRI concern skin conductance reactivity (SCR), heart rate variability (HRV), and respiration cycle (RC), in order to provide additional measures of physiological arousal that can explain part of the fMRI and PDR signal and in order to remove physiological noise from the functional MRI data.

Finally, assessment of childhood trauma (using the Childhood Trauma Questionnaire, short form (CTQ-SF)) [103] is performed at T0 to assess moderating effects on treatment success. At T1, the Helping Alliance Questionnaire-II (HAQ-II) [104] will be administered in the PCT condition to assess the role of therapeutic relationship in therapy success. At T1 and T2, the Brugha recent life events questionnaire [105] will be administered to obtain information on the occurrence of life events during the trial. All questionnaires have been validated and have shown good reliability. An overview of the assessments used per treatment phase is provided in Table 1.

**Table 1:** Overview of assessments

	Screening	Baseline T0	Follow-up T1 (3 month)	Follow-up T2 (18 month)
IDS-SR	x	x	x	x
SCID-I interview			x	
incl. Life chart method	x			x
DART	x			
ERQ		x	x	
LEIDS-RR		x	x	
LARSS		x	x	
RPA		x	x	
BVAQ		x	x	
CTQ		x		
NEO-FFI		x	x	
DAS		x	x	x
PANAS		x	x	
DDOPS		x	x	
ARDPEI with PDR		x	x	
IAT with PDR		x	x	
Emotional Reasoning Task		x	x	
WAIS-IV subtests		x	x	
fMRI ERT with PDR + SCR + HRV + RC		x	x	
fMRI VWMT with PDR + SCR + HRV + RC		x	x	
fMRI Resting State with SCR + HRV + RC		x	x	
MRI T1 with SCR + HRV + RC		x	x	
MRI arterial spin labelling with HRV + RC		x	x	
HAQ-II			x	
Brugha list			x	x

### Procedure

#### Overall procedure

All data will be collected at the University Medical Center Groningen, the Netherlands. Individuals interested in participation will contact the researchers on their own initiative, following public advertisement. During the screening, the researchers will first check if the participant fully understands the study, before the participant will sign an informed consent form. Then the SCID-I, IDS-SR, Dutch Adult Reading Test (DART) [106], an MRI checklist and a questionnaire with several socio-demographic background questions will be administered, all to confirm that the participant meets the inclusion criteria.

To minimize the burden on the day of scanning, a number of questionnaires will be sent to the participants 1 week prior to the baseline assessment. During baseline assessment the rest of the self-report questionnaires will be administered, the cognitive tests will be performed (ARDPEI and IAT, during which pupil dilation and gaze tracking will be measured with the Research Eyelink 1000 Eye tracker (Mississauga, Canada), plus the Emotional Reasoning Task and the WAIS-IV subtests), and finally participants will engage in an MRI-scanning session.

After baseline assessment, HC participants have finished their participation, and participants in the rrMDD group undergo either eight sessions of PCT, or are in the waiting list condition. Shortly after treatment (3 months following baseline), the first follow-up assessment T1 will be performed, in which the whole baseline procedure will be repeated (minus the CTQ and plus the HAQ-II and Brugha list). Eighteen months after baseline, a shortened version of the clinical assessment (SCID-I (including assessment of psychopathology since T0 with the life chart method), IDS-30 and DAS) will be repeated to assess stability of clinical state. Participants will receive 25 euro per assessment, 75 euro in total, plus reimbursement of travel expenses.

#### MRI procedure

MRI scanning will be performed on two scanners (due to an unforeseen scanner replacement). The 25 HC and the first 50 rrMDD subjects will be scanned on a Philips Intera 3 Tesla MR system, equipped with a 32-channel receiver head coil, at the NeuroImaging Center, University Medical Center Groningen. The last 25 rrMDD patients (all in the treatment condition) will be scanned on a Siemens 3 Tesla Magneton Prisma MR system (equipped with a 64-channel receiver head coil), at the Radiology Department of the University Medical Center Groningen, using imaging protocols harmonized to the Philips protocols.

The scanning procedure involves two functional echo planar imaging (EPI)-based acquisitions (TR/TE 2000/30 ms, 90° flip angle, voxel size 3.5 × 3.5 × 3.5 mm) to measure BOLD contrast during the ERT and the VWMT, one functional EPI-based acquisition (TR/TE 2000/30 ms, 70° flip angle, voxel size 3.5 × 3.5 × 3.5 mm) sensitive to BOLD contrast during rest (RS), one T1-weighted structural scan for anatomical reference (TR/TE 9/3.5 ms, 8° flip angle, voxel size 1x1x1mm), and finally a pseudo-continuous arterial spin labelling (pCASL) acquisition (TR/TE 2000/14 ms, 90° flip angle, voxel size 3x3x3).

During the ERT, VWMT and RS acquisitions, simultaneous pupillometry will be recorded using an SR-Research MR-compatible Eyelink system (Mississauga, Canada). Besides changes in brain activation and pupil dilation, autonomic responses to emotional events and stimuli include increased skin conductance reactivity (SCR) and changes in cardiovascular activity (HRV) [107]. We will measure SCR during MRI scanning using an MR-compatible Direct Current Galvanic Skin Response MR sensor interfaced with the BrainAmp ExG MR amplifier (Brain Products, GmbH) by applying a constant voltage (.5 V) between two sintered Ag/AgCl electrodes attached to the palmar surface of the distal phalanges of the index and middle fingers of the left hand. Furthermore, HRV signal will be recorded during scanning, logging the R-top trigger produced by the standard cardiac equipment of the Philips and Siemens MRI systems. In order to correct for additional noise, respiratory rate and depth will be measured through pressure variation in a cushion that is fastened around the participant’s abdomen. Finally, before every fMRI acquisition, the state tension levels of the participant will be monitored, by asking them how tense they feel on a Visual Analogue Scale.

#### Randomization

Allocation sequence will be based on computer-generated random numbers. The first 50 rrMDD patients will be randomized over either the treatment condition or the waiting list control condition, to allow for instantaneous analysis of immediate PCT effects after inclusion of these participants. The 25 last included rrMDD patients will be allotted to the treatment condition, but will be given the same information (and thus hold the same expectations regarding their chance of being in the treatment condition). Patients and the principal investigators will not be blind to the treatment condition. However, to ensure unbiased assessment of clinical state and neuropsychological testing, the researchers who are involved in further assessments will be kept blind, and participants will be asked not to inform the assessor on their allotted condition.

### Statistical analyses

#### Questionnaire and behavioural data

Cross-sectional residual characteristics of remitted MDD will be tested with a (Repeated Measures-, in case of highly correlated task conditions) AN(C) OVA procedure. The effects of PCT as measured with questionnaires and cognitive tests before and after treatment will be analysed within a multi-level analysis framework. Appropriate nonparametric tests (e.g. Friedman test) will be used if warranted. Effects will be considered significant at *p* < .05. Age, sex, and education level will be added as covariates.

#### MRI data

Quality of BOLD fMRI data will be extensively checked, before data will be pre-processed according to standard recommended procedures. Subsequently, data will be modelled on the subject level using onsets/duration for the different task conditions, or time-course information. On the second level, between-group comparisons will be performed in order to analyse residual abnormalities in emotion regulation capacity, verbal working memory performance and resting state-perfusion and functional connectivity. A multilevel analysis model will be set up to test for the effects of treatment on these measures. Furthermore, linear modelling will be applied to identify and test the predictors of long-term treatment success, as defined by symptomatology (at T0, T1, T2) and relapse status and course (T1-T2) in the larger sample of remitted patients who have received therapy. Multivariate pattern analysis will be performed to evaluate the predictive value of post-treatment characteristics and pre-treatment changes for long-term treatment success. Effects will be considered significant at *p* < .05, corrected for multiple comparisons.

#### Pupillometry data

Pupillometry data will be corrected for eye blinks and modelled to task data. Summary statistics will be entered in (Repeated Measures-)AN(C) OVAs, multi-level models and linear regression models. Effects will be considered significant at *p* < .05 after appropriate correction for multiple comparisons. To investigate whether PDR measurements have value for predicting frontal brain activation during emotion regulation, the PDR will be related to functional activation of regions implicated in effortful emotion regulation using multiple regression, while controlling for the arousal component of the sympathetic response, in the form of variation in SCR and HRV.

To investigate whether these relations are unique for effortful emotion regulation, linear regression models of the PDR during effortful emotion regulation (ERT) and during working memory (VWMT) will be set up and compared. Finally, it will be investigated whether treatment success can be predicted from multivariate patterns based on information from different modalities.

## Discussion

The high prevalence of recurrence in MDD poses a major clinical challenge and requires a better understanding of relapse vulnerability and of factors underlying preventive therapy success [108]. Recent reports explicitly call for combined neuroscientific and clinical research to improve current treatment [8, 109, 110]. The NEWPRIDE trial will be the first to study working mechanisms and predictors of Preventive Cognitive Therapy by examining neurophysiological and cognitive processes associated with attentional processing and regulation of both positive and negative emotional information.

In this RCT, we examine vulnerability for relapse by comparing pre-treatment neurocognitive processing in rrMDD with a group of HC, and we investigate hypothesized changes induced by PCT as compared to a waiting list control condition. Clinical, cognitive, and fMRI assessments in the remitted patient group are performed immediate and 15 months after treatment, to gain insight in the working mechanisms of preventive cognitive therapy and to examine predictors of relapse and relapse prevention.

One of the main strengths of the present study is the composition of the patient sample: only highly recurrent remitted MDD patients are included, allowing for the thorough examination of relapse mechanisms. Furthermore, given the expected relapse rate of 50% within 1,5 years follow-up, the relatively high recurrence in the present sample makes it possible to study predictors of (prevention of) relapse. The lack of confounding antidepressant medication use, recent cognitive therapy use, or current comorbid psychiatric diagnoses makes for a clean examination of residual characteristics and therapy effects. Another strength is the thorough investigation of clinical and neurocognitive features in this study, providing a broad and extensive investigation of mechanisms facilitating vulnerability and prevention of relapse.

A methodological difficulty of the follow-up design is the risk of participants dropping out, a risk enlarged by the expected amount of relapse. If possible, participants who drop out will be replaced. For the longitudinal analysis, the number of included participants will be sufficient to allow for an estimated 20% loss of participants and to detect an expected medium-sized within-group treatment effect. Furthermore, the fact that the last group of participants is scanned on a different MR-scanner might lead to higher between-group variance. Fortunately, the scanner change only affects the longitudinal analyses of PCT success prediction in the treatment condition. Since we anticipate a 50% relapse in both first-scanner and second-scanner participant group, and because pre- and post-treatment scanning is performed on the same scanner in all participants, we expect that any effect of the scanners will be equally divided between the relapse- and no-relapse groups, thereby minimizing possible limiting effects of the scanner change.

## Conclusion

In conclusion, by examining neurocognitive characteristics of rrMDD, the NEWPRIDE study will provide more insight in vulnerability to relapse and working mechanisms of psychological relapse prevention interventions. Unravelling the mechanisms of relapse prevention will improve our understanding of changes that are needed to lower an individual’s relapse vulnerability and may add to the development of more targeted and personalised interventions. Furthermore, results of the study may lead to the identification of neurocognitive predictors of both individual relapse risk and the chance that an individual might benefit from PCT, based on characteristics in the remitted phase. Finally, as routinely performing neuroimaging investigations for predicting treatment success is clinically not feasible, this study aims to validate the PDR as a marker of brain activation during emotion regulation in remitted MDD, for use in innovative non-imaging, brain-informed prediction and monitoring of PCT success.

## Data Availability

Data will be entered by two separate researchers, and anonymously stored on a shielded drive. Personal information will be stored separately in password-protected files. Only the authors have access to the final dataset. Analytical code and anonymised data will become available from the senior author on request. We will submit study results for publication in peer reviewed journals and presentation at (inter) national conferences. There are no publication restrictions. We will notify participants of publication.

## Abbreviations

ACC: anterior cingulate cortex
Ag: silver
AgCl: silver chloride
AN(C)OVA: analysis of (co)variance
ARDPEI: Attentional Response to Distal vs. Proximal Emotional Information
BOLD: blood oxygenation level dependent
BVAQ: Bermond-Vorst Alexithymia Questionnaire
CTQ: Childhood Trauma Questionnaire short form
DART: Dutch Adult Reading Test
DAS-A: Dysfunctional Attitude Scale form A
DDOPS: Domains and Dimensions of Pleasure Scale
EPI: echo planar imaging
ERQ: Emotion Regulation Questionnaire
ERT: Emotion Regulation Task
fMRI: functional magnetic resonance imaging
HAQ-II: Helping Alliance Questionnaire-II
HC: healthy control
HRV: heart rate variability
IAT: Implicit Association Task
IDS-SR: Inventory of Depressive Symptomatology – Self-Report
LARSS: Leuven Adaptation of the Rumination on Sadness Scale
LEIDS-RR: Leiden Index of Depression Sensitivity 2nd revision
MDD: Major Depressive Disorder
MDE: Major Depressive Episode
mm: millimeter(s)
MR: magnetic resonance
MRI: magnetic resonance imaging
ms: millisecond(s)
NEO-FFI: NEO Five Factor Inventory
NEWPRIDE: Neurocognitive Working Mechanisms of the Prevention of Relapse in Depression
NWO: Dutch Research Council
PANAS: Positive and Negative Affect Scale
pCASL: pseudo-continuous arterial spin labelling
PCT: Preventive Cognitive Therapy
PDR: pupil dilation response
PFC: prefrontal cortex
RC: respiration cycle
RCT: randomized controlled trial
rMDD: remitted Major Depressive Disorder
RPA: Responses to Positive Affect
rrMDD: remitted recurrent Major Depressive Disorder
RS: resting state
SCID-I: Structured Clinical Interview for DSM-IV Axis I disorders
SCR: skin conductance reactivity
T0: timepoint 0, baseline
T1: timepoint 1, 3-month follow-up
T2: timepoint 2, 18-month follow-up
TE: echo time
TR: repetition time
V: voltage
VWMT: Verbal Working Memory Task
WAIS-IV: Wechsler Adult Intelligence Scale-IV
